# Supplementary material for: The Use of Armeo®Spring Device to Assess the Effect of Trunk Stabilization Exercises on the Functional Capabilities of the Upper Limb—An Observational Study of Patients after Stroke
Source: Sensors (Basel). 2022 Jun 8;22(12):4336. doi: 10.3390/s22124336 (PMC9229540; doi:10.3390/s22124336)
Supplement: Supplementary file 1 [file sensors-22-04336-s001.zip › sensors-1730080-supplementary.pdf]

SUPPLEMENT—FIGURES and FIGURES CAPTIONS

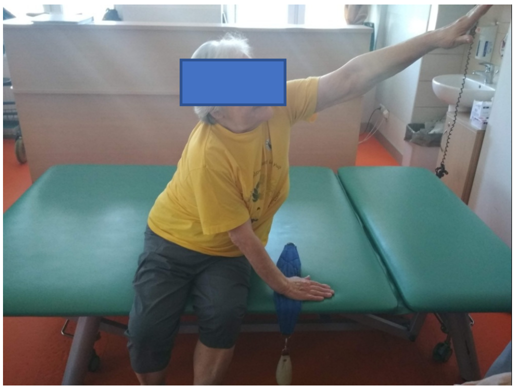

Figure S1. Exercise in the sitting position, taking into account the rotation of the trunk (counter-rotation of the girdle), and the hands of the indirect and directly affected limb resting on the therapeutic table. The directly affected limb cannot tear itself away from the table. (source: own collection)

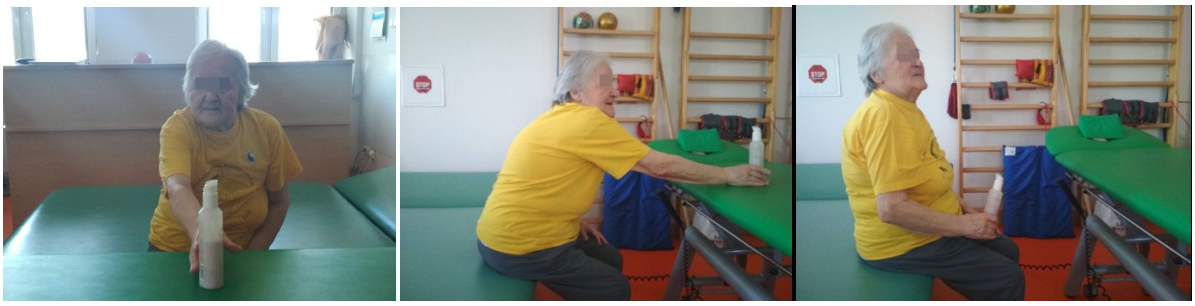

(a) (b) (c)

Figure S2. Exercise in the sitting position of the directly affected limb. Front view (a), side view while reaching (b), and after completing the task (c). The patient's task is to reach for an object placed at a certain distance in front of the patient using only the directly affected limb. (source: own collections)

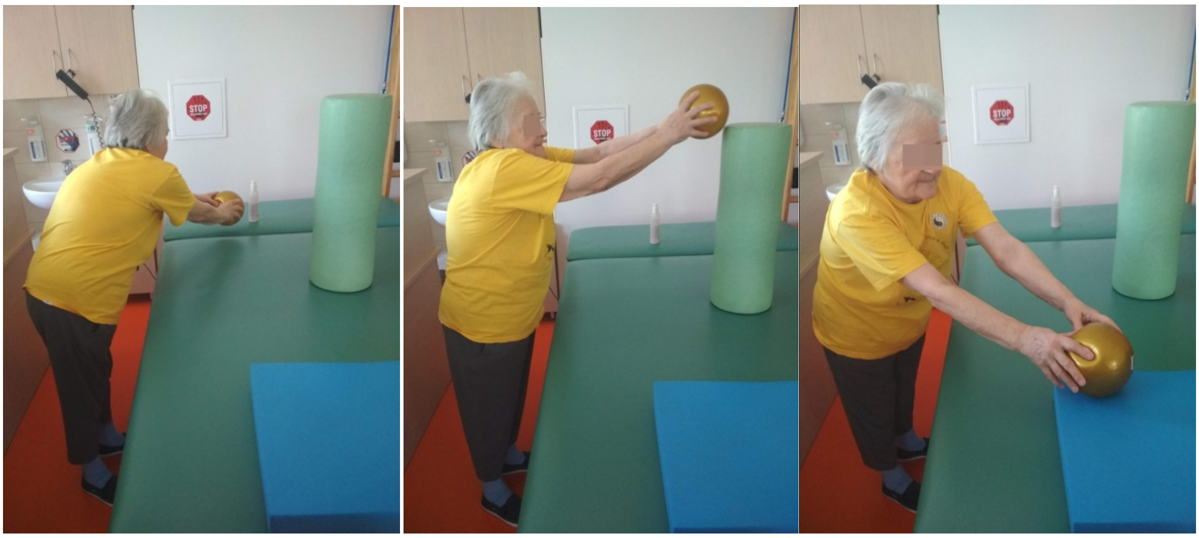

(a) (b) (c)

Figure S3. Exercise at the activity level according to ICF classification - reaching the designated point on the left side, in front and on the right side with both hands (a–c), crossing the midline of the body, trunk rotation. (source: own collection)

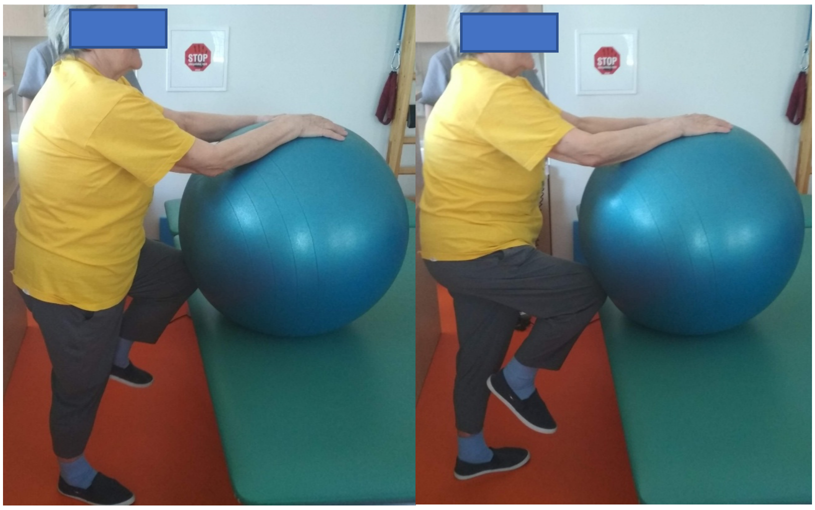

(a) (b)

Figure S4. (a,b) Alternately reaching with the knees to a large ball with the directly affected limb and indirectly affected, stabilized trunk, upper limbs on unstable ground (source: own collection)
